# Nonalcoholic Fatty Liver Disease in Children and Adolescents Taking Atypical Antipsychotic Medications: Protocol for a Systematic Review and Meta-analysis

**DOI:** 10.2196/20168

**Published:** 2022-03-21

**Authors:** Reem Hatem, Faisal A Nawaz, Ghadah A Al-Sharif, Mohammad Almoosa, Wid Kattan, Christos Tzivinikos, E Lila Amirali, Ammar Albanna

**Affiliations:** 1 College of Medicine Mohammed Bin Rashid University of Medicine and Health Sciences Dubai United Arab Emirates; 2 Division of Psychiatry, Department of Medicine College of Medicine King Abdulaziz University Jeddah Saudi Arabia; 3 Al Jalila Children's Specialty Hospital Dubai United Arab Emirates; 4 Department of Psychiatry and Addiction Université de Montréal Montréal, QC Canada

**Keywords:** nonalcoholic fatty liver disease, psychopharmacology, antipsychotics, children, adolescents, overprescribing, pharmaceuticals, antipsychotic medications, medication, pediatric psychopharmacology, pharmacology, child and adolescent psychiatry

## Abstract

**Background:**

Atypical antipsychotics (AAP) are commonly prescribed to children and adolescents and are associated with important adverse effects including weight gain and metabolic syndrome. Nonalcoholic fatty liver disease (NAFLD) is not only the most common pediatric liver disease but is also associated with serious complications including liver cirrhosis.

**Objective:**

Given that NAFLD and AAP are associated with metabolic syndrome, we aim to comprehensively examine the association between AAP and NAFLD in children and adolescents.

**Methods:**

We will conduct a systematic review of studies exploring NAFLD in subjects younger than 18 years on AAP published in English between 1950 and 2020 following the PRISMA (Preferred Reporting items for Systematic Reviews and Meta-Analysis) guidelines.

**Results:**

A PRISMA flowchart will be used present the study results after comprehensively reviewing studies on NAFLD in children and adolescents taking AAP. The first and second systematic searches will be conducted during December 2021. The results are expected to be published in June 2022.

**Conclusions:**

This research project will serve as a foundation for future studies and assist in devising interventions and reforming clinical guidelines for using AAP to ensure improved patient safety.

**International Registered Report Identifier (IRRID):**

PRR1-10.2196/20168

## Introduction

There is an increasing trend in prescribing atypical antipsychotics (AAP) to children and adolescents [1], and this merits a better understanding of their long-term adverse effects. Metabolic complications such as weight gain, hyperlipidemia, and insulin resistance are known side effects of AAP [2-4], and studies have suggested that youngsters are at an increased risk of these side effects compared to older adults [5]. With the decreased incidence of typical psychotic use in children and adolescents it is reasonable to speculate that children on AAP may be at a greater risk of disorders associated with metabolic syndrome, including nonalcoholic fatty liver disease (NAFLD) [6-9].

NAFLD in children and adolescents younger than 18 years is the most common cause of pediatric liver disease, with a prevalence of approximately 9.6% [10]. The prevalence significantly increases to approximately 77% among obese children [11], suggesting its association with metabolic syndrome [12]. NAFLD comprises a spectrum of liver diseases that range from the accumulation of fat in hepatocytes (steatosis) and inflammatory liver changes including nonalcoholic steatohepatitis (NASH) to more serious complications such as liver cirrhosis [13,14]. The “two-hit” theory attempts to explain the pathogenesis of NAFLD [15]; the first hit is due to the accumulation of triglycerides in hepatocytes causing liver steatosis, and the second hit is attributable to many factors including oxidative stress and increased cytokines leading to inflammatory changes.

NAFLD in children and adolescents is associated with serious long-term adverse outcomes, including increased risk of mortality and liver cirrhosis requiring transplantation [16]. Multiple risk factors and medical conditions including eating disorders have been linked to the etiology of NAFLD [17,18]. Obesity has been consistently identified as an important risk factor for pediatric NAFLD [11]. Insulin resistance is another risk factor for NAFLD; Nobili et al prospectively followed 84 children with NAFLD and reported that they were almost always insulin-resistant regardless of their BMI [19]. Moreover, a sedentary lifestyle and high fructose consumption have been described as risk factors for NAFLD [20,21]. Furthermore, epidemiological studies have identified being male, older age, and Hispanic ethnicity as risk factors for childhood NAFLD [10]. Additionally, genetic, cellular, and hormonal factors [22] have been found to influence the transition to inflammatory hepatic changes.

Liver biopsy is the gold standard diagnostic test for NAFLD [23,24], although it is an invasive procedure that can be associated with serious complications. Liver function tests (LFTs) are among the first-line investigations for NAFLD [25,26]. However, the use of LFT to diagnose NAFLD is challenging due to the low sensitivity of this method and the discrepancy around the appropriate cutoff values [13]. Moreover, the normal LFT level does not exclude the presence of advanced NAFLD. In contrast, hepatic ultrasonography (US) is a safe, noninvasive, and widely available imaging tool for the assessment of NAFLD. In adults, it has acceptable sensitivity and specificity (100% and 90%, respectively), especially when the liver fat percentage exceeds 20% [27]. Similar statistical properties were documented in a pediatric study [28]. Other radiological diagnostic tools for NAFLD include computed tomography (CT), magnetic resonance imaging (MRI), and magnetic imaging spectroscopy (MRS) [29].

There is limited information regarding the risk of NAFLD in children and adolescents treated with AAP despite their association with metabolic complications that are considered risk factors for the development of NAFLD [30,31]. The objective of our study is to conduct a comprehensive systematic review of the available literature to examine the association between AAP use and NAFLD in children and adolescents.

## Methods

### Study Identification

To capture all relevant literature on NAFLD among children and adolescents on AAP, we plan to conduct 2 systematic literature reviews. In our first search, we aim to identify studies that assess NAFLD in children and adolescents taking AAP. Given that we expect a paucity in such studies based on a pilot search performed, we will include studies of different designs including cohort and case-control studies, case reports, and case series. In our second systematic search, we will comprehensively review AAP trials in children and adolescents, attempting to identify any reports of NAFLD; we intend to determine whether this was documented as a primary outcome, a secondary outcome, or as an incidental finding in these studies.

Our systematic review will be performed according to this predefined protocol that describes the objectives, search strategy, eligibility criteria, and evaluation methods according to the PRISMA (Preferred Reporting items for Systematic Reviews and Meta-Analysis) guidelines [32].

### Systematic Review Methodology

We will conduct 2 systematic literature reviews. The search is restricted to studies published in English from January 1, 1950, until March 31, 2021. We selected 1950 as the starting year for the literature search, as it coincides with the development of the first antipsychotic.

In our first search, we aim to identify studies that assess NAFLD in children and adolescents taking AAP. We will use the following search terms: “second-generation neuroleptics,” antipsychotics,” and “neuroleptics,” with their generic and brand names given in Table 1. Further, the following variations of pediatric NAFLD, namely “NAFLD,” “NASH,” “nonalcoholic fatty liver disease,” “nonalcoholic steatohepatitis,” “hepatic steatosis,” “fatty liver disease,” “nonalcoholic fatty liver,” and “fatty liver.” In our second search, we will include the following keywords: “atypical antipsychotics” and “atypical neuroleptics” [33].

**Table 1:** Generic and brand names of all the atypical antipsychotics in the inclusion criteria of our first and second literature searches.

Medication name	Brand name(s)
Amisulpride	Amazeo, Amipride, Amival, Solian, Soltus, Sulpitac, Sulprix, Midora, Socian
Aripiprazole	Abilify, Abilify Maintena, Abilicare, Abilia, Abelfiz, Abdin, Abizol, Abyraz, Aceprofen, Adexyl, Adwiprazole, Alcartis, Alembic, Pipzol, Amdoal, Anasil, Andepro, Antredamin, Ao Pai, Apalife, Apaloz, Apipral, Apiprax, Apra, Aprizexen, Arena, Arepexane, Aria, Aribit, Aricogan, Arifay, Arileto, Arilan, Arilex, Arimed, Aripa, Aripat, Aripegis, Aripen, AripiHexal, Aripilek, Aripip, Aripipa, Aripipan, Aripiprazol, Aripiprex, Aripizin, Ariple, Ariply, Arip-MT, Aripra, Ariprax, Ariprazole, Ariprexa, Ariprizol, Aripsan, Ariski, Arisppa, Aristab, Arive, Arives, Arixind, Arize, Arizol, Arlemide, Arpilif, Arpit, Arpizol, Arpoya, Arypiprazol, Glenmark, Aryzalera, Arzip, Arzu, Asduter, Asprito, Astoret, Atfren, Azolar, Azymol, Bipodis, Brisking, Centalify, Confilify, Curexol, Egisazol, Epimate, Explemed, Fixment, Gemplex, Ignis, Ilimit, Ipipral, Irazem, Kavium, Lazurex, Lemidal, Lemilvo, Madepzol, Motruxia, Neoaripi, Oryva, Otsuka Albilify, Parokzol, Paxifor, Pipra-A, Piprason, Prazarit, Rapiproz, Real One, Restigulin, Rima-Fix, Ripazol, Sayfren, Schizofy, Schizopra, Sensaz, Siblix, Siznil, Sizopra, Tevaripiprazole, Trefero, Zolerip, Zolprix, Zydus, Zykalor, Zylaxera, Aristada
Asenapine	Saphris, Sycrest
Blonanserin	Lonasen
Cariprazine	Reagila, Reagyla, Vraylar
Clozapine	Clozaril, Clopine, Clozapine Synthon, Versacloz
Iloperidone	Fanapt, Zomaril
Lurasidone	Latuda, Lusiax, Luradon, Lurap, Lurasidone Hydrochloride, Lustona
Melperone	Bunil, Buronil, Melneurin, Eunerpan
Olanzapine	Abilanz, Absolute, ACT Olanzapine, Aedon, Amulsin, Anzap, Anzatric, Anzorin, Apo Olanzapine, Apzet, Arenbil, Arkolamyl, As-Pineks, Auro Olanzapine, Axonium, Aziva, Balerap, Bloonis, Caprilon, Cap Tiva, Chemmart Olanzapine, Crisapina, Deprex, Domus, Dopin, Dozic, Egolanza, Elynza, Enolex, Epilanz-10, Expolid, Exzapine, Ferzapin, Fontanivio, Fredilan, Irropia, Jamp Olanzapine, Jolyon MD, Joyzol, Ketoconazol Sesderma, Kotico, Kozylex, Lanopin, Lanzafen, Lanzapine, Lanzek, Lanzek Zydis, Lapenza, Lapin, Lapozan, Lazap, Lazapix, Lezapin-MD, Lopez, Lupilan, Malanxin, Manza, Marathon, Marcato, Mar-Olanzapine, Medlanz, Meflax, Meltolan, Midax, Mint Olanzapine, MylanOlanzapine, Nervix, Neupine, Newzypra, Nodoff, Norpen OroNykob, Nyzol, Oceanil, Ofans ODT, Oferta, Oferta Sanovel, Olace, Oladay, Olafer, Olafid,Olan, Olanap, Olandix, Olandoz, Olandus, Olanex, Olanexyn, Olanpax, Olansapiin Mylan, Olansek, Olanstad, Olanz, Olanza, Olanzacor, Olanzalet, Olanzalux, Olanzamed, Olanzapin, Olanzapro, Olanzar, Olanzavitae, Olanzep, Olanzin Olanzyl, Olapex, Olapin, Olapine, Olaprexa, Olastazen, Olavex, Olaxinn, Olazap, Olazax, Olazin, Olazine, Olazofren, Oleanz, Olenxa, Olexar, Olfrex, Olivin, Ollafax, Olmed, Olmyzem, Olnegis, Olpax, Olpin, Olpinat, Oltal, Olza, Olzadin, Olzanid, Olzap, Olzapin, Olzic, Olzin, Onezyp, Onotran, Onza, Onzapin, Opin, Opirap, Ou Lan Ning, Ozapex, Ozapin-MD, Ozapram, Ozaprin, Ozin, Parnasan, Parnassan, Pericam, Pinolza, Placet, PMS-Olanzapine, Polar, Pranza, Prexal, Prexolan, Prolanz, Protil, Pryzex, Psychozap, Ranofren, RAN-Olanzapine, Redilanz, Remital, Revertrix, Rexapin, Rexepi, Rolanzax, Sartina, Simina, Sincris, Sizap, Solazin, Stygapon, Synza, Tolaz, Treana, Trexol, Vaincor, Vaira, Villamos, Ximin, Xoltiva, Xytrex, Zalasta, Zalepin, Zanprex, Zap, Zapiluks, Zapilux, Zapin, Zapinex FT, Zappa, Zaprinel, Zapris, Zelta, Zeprex, Zesten, Ziora, Zirmapina, Zofrenix, Zolafren, Zolamelt, Zolapine, Zolaswift, Zolaxa, Zonapin, Zophix, Zopix, Zopridoxin, Zoxil, Zylanza, Zypadhera, Zypeace, Zypine, Zyprexa, Zyzapin, Zypadhera, Zyprexa Relprevv
Paliperidone	Aspire-XR, Inveda, Invega, Palido, Parnido, Trevicta, Xeplion
Quetiapine	Actawell, Adequet, Aebol, Afidat, Aretaeus, Asicot, As-Kalmeks, Atip, Atrolak XL, Biquelle XL, Biquetan, Bonogren, Brancico XL, Catepsin, Cizyapine, Dopaquel, Equelib, Esertia, Etiagen XR, Etipin, Geldoren, Gofyl, Hedonin, Keday XR, Kenantis, Ketap, Ketidose, Ketilept, Ketinel, Ketipina, Ketipine, Ketipinor, Ketrel, Kvelux, Kventiax, Kvineva, Kwetaplex XR, Loquen, Mintreleq XL PR, Mylan Quetiapine, Nantarid, Norsic, Pincalm, Pinexet, Placidin, Psynil, Psyquel, Psyquet, Q-Mind, Q-Pin, Qpine, Quantia, Queapin, Quel, Quentiax, Queopine, Quepigal, Quepimax, Queropax, Quetap, Queteper, Quetia, Quetiapina, Quetiaros, Quetiazic, Quetidin, Quetimax, Quetin, Quetipax, Quetipin, Quetirel, Quetium, Quetkare, Quetoser, Quitapex, Qurax, Quser, Qutace, Qutan, Quticool, Qutipin, Q-Win, Sequa, Serenase, Serex, Seroquel, Setinin, Sizonorm, Sizoquit, Socalm, Sofrel, Symquel XR, Tevaquel, Tiapinan, Tiapine, Tomel, Treksta, Valir, Vesparax, Volqer, Vorta, Alcreno, Alzen, Anaquetan XR, ApoTiapina, Arezil XR, Asicot, Atrolak, Biatrix, Biquetan, Brevenox, Cacepin, Calm-ez, Cedrina, Centroqueen, Delucon, Dendritex, Derin, Derin Prolong, Dominium, Edagan, Etiaben XR, Etiagen, Etiapin, Etiasel, Eufrenin, Geroquel, Gofyl, Gyrex, Hedonin, Hiloca, Ilufren, Inquetia, Kagitz, Kalm, Kaptan, Kefrenex, Kemoter, Kesaquil, Ketian XR, Ketiap, Ketilept, Ketipine, Ketipinor, Ketya, Kvelux, Kventiax, Kvetiapin, Kwetaplex, Kwetax, Limus, Loquen, Loquen XR, Matepil, Megazone, Nantarid, Netiapin, Neuroquel, Neutapin, Psicotric
Risperidone	Risperdal, Risperdal Consta, Risperdal M-Tab, Risperdal Quicklets, Risperlet
Sertindole	Serdolect, Serlect
Sulpiride	Dogmatil, Dolmatil, Eglonyl, Espiride, Modal, Prometar, Sulpor
Ziprasidone	Geodon, Pramaxima, Vikolus, Ypsila, Zeldox, Zipradon, Zipragen, Zipramyl, Ziprasidon, Zipsydon, Zipwell, Zypsila, Zypsilan, Zeldox
Zotepine	Losizopilon, Lodopin, Setous, Zoleptil

Medications in combination with Fluoxetine will not be included in the research (ie, Olanzapine: Co-Depricap, Depten-OZ,Rexepi Combi,Symbyax, Tagram, Target, Olapin Forte, Olapin Plus,Olanex F, Oladay-F).

In our second systematic search, we will comprehensively review AAP trials in children and adolescents, attempting to identify any reports of NAFLD; we aim to determine whether this was documented as a primary outcome, a secondary outcome, or as an incidental finding during these studies. The following databases will be used to conduct the search: Ovid MEDLINE, Cochrane, and Cumulative Index to Nursing and Allied Health Literature (CINAHL). We also searched the following databases up to 2016: Embase, Web of Science, BIOSIS Previews, and PsycINFO.

A systematic, computer-assisted search of the following databases will be performed by 4 medical students RH, FN, GA, and MA at the Mohammed Bin Rashid University (MBRU): Ovid MEDLINE, Embase, Web of Science, BIOSIS Previews, PsycINFO, and CINAHL. The search strategy will be supported by librarian Shakeel Tegginmani at the MBRU Library. Furthermore, the bibliographies of the retrieved and relevant articles will be manually searched for relevant articles. Full articles in English published in peer-reviewed journals will be included in this review.

As mentioned earlier, we will conduct 2 searches that will be limited to children and adolescents younger than 18 years of age. The first search will include the following variations of AAP: “atypical antipsychotics,” “atypical neuroleptics,” “second-generation antipsychotics,” “second-generation neuroleptics,” “antipsychotics,” and “neuroleptics” for AAP [34].

### Eligibility Criteria

We determined the following inclusion and exclusion criteria based on a literature review and the objectives of this study. All medication trials published in English pertaining to AAP in subjects younger than 18 years of age that reported NAFLD as an outcome, assessed by radiological methods (including liver ultrasound) or liver biopsy, will be included in this review. Moreover, prospective and retrospective observational studies of children and adolescents on AAP with reported NAFLD indicators will be included. Finally, case series and reports of NAFLD in children and adolescents on AAPs will be included. We will exclude conference abstracts, editorials, letters to editors, treatment guidelines, and studies published as abstracts only. Reviews (systematic and nonsystematic) will be excluded; however, the bibliographies of relevant papers will be reviewed. We will exclude studies in which AAP were used as an add-on or in combination with other medications (such as antiepileptics, first-generation antipsychotics, and selective serotonin uptake inhibitors), and medications that are a combination of AAP and selective serotonin uptake inhibitors including Fluoxetine; however, we will include studies where AAP constitute 1 arm of the study [35]. Due to the overlap of NAFLD risk factors with other comorbidities and varying nutritional backgrounds, we will also exclude studies examining patients with pre-existing medical conditions including eating disorders [17,18]. Our search will be limited to studies published in English, and nonhuman studies will be excluded. The review and comparison of results will be conducted using Endnote.

### Outcomes

The primary outcome of the present study is NAFLD in children and adolescents, as assessed by either liver biopsy or using a radiological tool including hepatic US, MRI, MRS, and transient elastography among children and adolescents on AAP. Despite the relationship between NAFLD and metabolic syndrome, we did not include variables of metabolic syndrome in our outcomes because it is not the focus of our review and it has been previously studied, including in a recent meta-analysis [36]. Secondary outcomes will include changes in liver enzymes.

### Data Extraction

Investigators RH, FAN, GAA, and MA will independently review the titles and abstracts of the retrieved studies and exclude duplicates and irrelevant studies based on the aforementioned eligibility criteria. All the potentially eligible abstracts will be further assessed for eligibility by thoroughly reviewing their full texts. Results will be compared, and discrepancies will be resolved by consensus and by consulting investigators AA, CT, and ELA when needed. The Cohen κ will be calculated as a measure of interrater agreement. All studies that meet the eligibility criteria will be included in our analyses. Data extraction will be carried out using a standard form.

The quality of the studies will be assessed using the STROBE (Strengthening the Reporting of Observational studies in Epidemiology) guidelines [37] for cohort, case-control, and cross-sectional studies and the Newcastle-Ottawa Scale for nonrandomized studies. The quality of randomized controlled trials will be evaluated using the GRADE (Grading of Recommendations, Assessment, Development and Evaluations) method.

If the results allow for conducting a meta-analysis, we will report the outcomes by calculating the weighted pooled estimate of changes in the outcomes [38]. The risk of bias will be evaluated using three types of homogeneity tests: (1) forest plot, (2) Cochrane Q test (chi-square test), and (3) Higgins I2 statistics. In the forest plot, greater overlap between the CIs indicates greater homogeneity.

## Results

The search results will be presented using the PRISMA flowchart. This study will comprehensively review literature pertaining to NAFLD in children and adolescents taking AAP.

The first and the second systematic searches will be conducted during December 2021. The title and abstract review will be performed by RH, FAN, GAA, and MA between December 1 and December 15, 2021. Further, the full-text review will be performed by the same researchers between December 15 and December 25, 2021. The results are expected to be published in June 2022. The results of this study may inform clinical guidelines for AAP use in children and adolescents.

## Discussion

### Clinical Significance

Studies have suggested that the pediatric population on AAP is more at risk of developing long-term adverse effects due to AAP, including weight gain, hyperlipidemia, and insulin resistance [14]. Therefore, we have grounds to speculate that children on AAP may be at a greater risk of developing disorders associated with metabolic syndrome, including NAFLD. This research project is specifically important due to the current trends in overprescribing AAP in children aged below 18 years [39].

Due to the dearth of systematic reviews on this vital topic, the need for understanding the possible association between AAP and NAFLD in this population across the literature landscape is warranted. Conducting this systematic review would serve as a comprehensive foundation for future studies and help devise interventions for the child and adolescent population.

### Conclusions

The PRISMA flowchart was chosen to present the results of this study. There is a global rise in the use of AAP in treating children and adolescents below the age of 18 [39], which can cause long-term adverse effects and metabolic complications. We hope this research project will serve as a foundation for future studies and assist in devising interventions and reforming clinical guidelines of AAP use for improved patient safety.

## Abbreviations

AAP: atypical antipsychotics
LFT: liver function test
MRI: magnetic resonance imaging
MRS: magnetic resonance spectroscopy
NAFLD: nonalcoholic fatty liver disease
PRISMA: Preferred Reporting items for Systematic Reviews and Meta-Analysis
US: ultrasonography

## References

[1] Pringsheim T, Lam D, Patten SB (2011). The pharmacoepidemiology of antipsychotic medications for Canadian children and adolescents: 2005–2009. J Child Adolesc Psychopharmacol.

[2] Fedorowicz VJ, Fombonne E (2005). Metabolic side effects of atypical antipsychotics in children: a literature review. J Psychopharmacol.

[3] Perez-Iglesias R, Vazquez-Barquero JL, Amado JA, Berja A, Garcia-Unzueta MT, Pelayo-Terán JM, Carrasco-Marín E, Mata I, Crespo-Facorro B (2008). Effect of antipsychotics on peptides involved in energy balance in drug-naive psychotic patients after 1 year of treatment. J Clin Psychopharmacol.

[4] Reynolds GP, Kirk SL (2010). Metabolic side effects of antipsychotic drug treatment–pharmacological mechanisms. Pharmacol Ther.

[5] Hammerman A, Dreiher J, Klang SH, Munitz H, Cohen AD, Goldfracht M (2008). Antipsychotics and diabetes: an age-related association. Ann Pharmacother.

[6] Erdogan A, Atasoy N, Akkurt H, Ozturk D, Karaahmet E, Yalug I, Yalug K, Ankarali H, Balcioglu I (2008). Risperidone and liver function tests in children and adolescents: a short-term prospective study. Prog Neuropsychopharmacol Biol Psychiatry.

[7] Erdogan A, Karaman MG, Ozdemir E, Yurteri N, Tufan AE, Kurcer MA (2010). Six months of treatment with risperidone may be associated with nonsignificant abnormalities of liver function tests in children and adolescents: a longitudinal, observational study from Turkey. J Child Adolesc Psychopharmacol.

[8] Karaman MG, Erdoğan A, Tufan E, Yurteri N, Ozdemir E, Ankarali H (2011). Liver function tests in children and adolescents receiving risperidone treatment for a year: a longitudinal, observational study from Turkey. Int J Psychiatry Clin Pract.

[9] Patel NC, Crismon ML, Hoagwood K, Johnsrud MT, Rascati KL, Wilson JP, Jensen PS (2005). Trends in the use of typical and atypical antipsychotics in children and adolescents. J Am Acad Child Adolesc Psychiatry.

[10] Schwimmer JB, Deutsch R, Kahen T, Lavine JE, Stanley C, Behling C (2006). Prevalence of fatty liver in children and adolescents. Pediatrics.

[11] Chan DFY, Li AM, Chu WCW, Chan MHM, Wong EMC, Liu EKH, Chan IHS, Yin J, Lam CWK, Fok TF, Nelson EAS (2004). Hepatic steatosis in obese Chinese children. Int J Obes Relat Metab Disord.

[12] Fu J, Shi H, Liu L, Jiang P, Liang L, Wang C, Liu X (2011). Non-alcoholic fatty liver disease: an early mediator predicting metabolic syndrome in obese children?. World J Gastroenterol.

[13] Vajro P, Lenta S, Socha P, Dhawan A, McKiernan P, Baumann U, Durmaz O, Lacaille F, McLin V, Nobili V (2012). Diagnosis of nonalcoholic fatty liver disease in children and adolescents: position paper of the ESPGHAN Hepatology Committee. J Pediatr Gastroenterol Nutr.

[14] Holtmann M, Kopf D, Mayer M, Bechtinger E, Schmidt MH (2003). Risperidone-associated steatohepatitis and excessive weight-gain. Pharmacopsychiatry.

[15] AlKhater SA (2015). Paediatric non-alcoholic fatty liver disease: an overview. Obes Rev.

[16] Bhala N, Angulo P, van der Poorten D, Lee E, Hui JM, Saracco G, Adams LA, Charatcharoenwitthaya P, Topping JH, Bugianesi E, Day CP, George J (2011). The natural history of nonalcoholic fatty liver disease with advanced fibrosis or cirrhosis: an international collaborative study. Hepatology.

[17] Yasutake K, Kohjima M, Kotoh K, Nakashima M, Nakamuta M, Enjoji M (2014). Dietary habits and behaviors associated with nonalcoholic fatty liver disease. World J Gastroenterol.

[18] Zhang J, Abbasi O, Malevanchik L, Mohan N, Denicola R, Tarangelo N, Marzio DH (2017). Pilot study of the prevalence of binge eating disorder in non-alcoholic fatty liver disease patients. Ann Gastroenterol.

[19] Nobili V, Marcellini M, Devito R, Ciampalini P, Piemonte F, Comparcola D, Sartorelli MR, Angulo P (2006). NAFLD in children: a prospective clinical-pathological study and effect of lifestyle advice. Hepatology.

[20] Nseir W, Hellou E, Assy N (2014). Role of diet and lifestyle changes in nonalcoholic fatty liver disease. World J Gastroenterol.

[21] Jin R, Welsh JA, Le N, Holzberg J, Sharma P, Martin DR, Vos MB (2014). Dietary fructose reduction improves markers of cardiovascular disease risk in Hispanic-American adolescents with NAFLD. Nutrients.

[22] Hassan K, Bhalla V, El Regal ME, A-Kader HH (2014). Nonalcoholic fatty liver disease: a comprehensive review of a growing epidemic. World J Gastroenterol.

[23] Kleiner DE, Brunt EM, Van Natta M, Behling C, Contos MJ, Cummings OW, Ferrell LD, Liu Y, Torbenson MS, Unalp-Arida A, Yeh M, McCullough AJ, Sanyal AJ, Nonalcoholic Steatohepatitis Clinical Research Network (2005). Design and validation of a histological scoring system for nonalcoholic fatty liver disease. Hepatology.

[24] Mann JP, Goonetilleke R, McKiernan P (2015). Paediatric non-alcoholic fatty liver disease: a practical overview for non-specialists. Arch Dis Child.

[25] Kumra S, Herion D, Jacobsen LK, Briguglia C, Grothe D (1997). Case study: risperidone-induced hepatotoxicity in pediatric patients. J Am Acad Child Adolesc Psychiatry.

[26] Copur M, Erdogan A (2011). Risperidone rechallenge for marked liver function test abnormalities in an autistic child. Recent Pat Endocr Metab Immune Drug Discov.

[27] Dasarathy S, Dasarathy J, Khiyami A, Joseph R, Lopez R, McCullough AJ (2009). Validity of real time ultrasound in the diagnosis of hepatic steatosis: a prospective study. J Hepatol.

[28] Shannon A, Alkhouri N, Carter-Kent C, Monti L, Devito R, Lopez R, Feldstein AE, Nobili V (2011). Ultrasonographic quantitative estimation of hepatic steatosis in children With NAFLD. J Pediatr Gastroenterol Nutr.

[29] Lee SS, Park SH (2014). Radiologic evaluation of nonalcoholic fatty liver disease. World J Gastroenterol.

[30] Manco M, Marcellini M, Devito R, Comparcola D, Sartorelli MR, Nobili V (2008). Metabolic syndrome and liver histology in paediatric non-alcoholic steatohepatitis. Int J Obes (Lond).

[31] Lui SY, Tso S, Lam M, Cheung EFC (2009). Possible olanzapine-induced hepatotoxicity in a young Chinese patient. Hong Kong Med J.

[32] Liberati A, Altman DG, Tetzlaff J, Mulrow C, Gøtzsche PC, Ioannidis JPA, Clarke M, Devereaux PJ, Kleijnen J, Moher D (2009). The PRISMA statement for reporting systematic reviews and meta-analyses of studies that evaluate health care interventions: explanation and elaboration. J Clin Epidemiol.

[33] Lauressergues E, Staels B, Valeille K, Majd Z, Hum DW, Duriez P, Cussac D (2010). Antipsychotic drug action on SREBPs-related lipogenesis and cholesterogenesis in primary rat hepatocytes. Naunyn Schmiedebergs Arch Pharmacol.

[34] Oh K, Park J, Lee SY, Hwang I, Kim JB, Park T, Lee H, Koo S (2011). Atypical antipsychotic drugs perturb AMPK-dependent regulation of hepatic lipid metabolism. Am J Physiol Endocrinol Metab.

[35] Bonhaus DW, Weinhardt KK, Taylor M, DeSouza A, McNeeley PM, Szczepanski K, Fontana DJ, Trinh J, Rocha CL, Dawson MW, Flippin LA, Eglen RM (1997). RS-102221: a novel high affinity and selective, 5-HT2C receptor antagonist. Neuropharmacology.

[36] Almandil NB, Liu Y, Murray ML, Besag FMC, Aitchison KJ, Wong ICK (2013). Weight gain and other metabolic adverse effects associated with atypical antipsychotic treatment of children and adolescents: a systematic review and meta-analysis. Paediatr Drugs.

[37] da Costa BR, Cevallos M, Altman DG, Rutjes AWS, Egger M (2011). Uses and misuses of the STROBE statement: bibliographic study. BMJ Open.

[38] Ahn E, Kang H (2018). Introduction to systematic review and meta-analysis. Korean J Anesthesiol.

[39] Harrison JN, Cluxton-Keller F, Gross D (2012). Antipsychotic medication prescribing trends in children and adolescents. J Pediatr Health Care.

